# Graded and pan-neural disease phenotypes of Rett Syndrome linked with dosage of functional MeCP2

**DOI:** 10.1007/s13238-020-00773-z

**Published:** 2020-08-27

**Authors:** Xiaoying Chen, Xu Han, Bruno Blanchi, Wuqiang Guan, Weihong Ge, Yong-Chun Yu, Yi E. Sun

**Affiliations:** 1 Shanghai Institute of Stem Cell Research and Clinical Translation, Shanghai East Hospital, Tongji University School of Medicine, Shanghai, 200120 China; 2 State Key Laboratory of Medical Neurobiology and MOE Frontiers Center for Brain Science, Institutes of Brain Science, Jing’an District Centre Hospital of Shanghai, Fudan University, Shanghai, 200032 China; 3 Department of Psychiatry and Biobehavioral Sciences, David Geffen School of Medicine, UCLA, Los Angeles, CA 90095 USA

**Keywords:** MeCP2, Rett Syndrome, human pluripotent stem cell, neural differentiation

## Abstract

Rett syndrome (RTT) is a progressive neurodevelopmental disorder, mainly caused by mutations in MeCP2 and currently with no cure. We report here that neurons from R106W MeCP2 RTT human iPSCs as well as human embryonic stem cells after MeCP2 knockdown exhibit consistent and long-lasting impairment in maturation as indicated by impaired action potentials and passive membrane properties as well as reduced soma size and spine density. Moreover, RTT-inherent defects in neuronal maturation could be pan-neuronal and occurred in neurons with both dorsal and ventral forebrain features. Knockdown of MeCP2 led to more severe neuronal deficits as compared to RTT iPSC-derived neurons, which appeared to retain partial function. Strikingly, consistent deficits in nuclear size, dendritic complexity and circuitry-dependent spontaneous postsynaptic currents could only be observed in MeCP2 knockdown neurons but not RTT iPSC-derived neurons. Both neuron-intrinsic and circuitry-dependent deficits of MeCP2-deficient neurons could be fully or partially rescued by re-expression of wild type or T158M MeCP2, strengthening the dosage dependency of MeCP2 on disease phenotypes and also the partial function of the mutant. Our findings thus reveal stable neuronal maturation deficits and unexpectedly, graded sensitivities of neuron-inherent and neural transmission phenotypes towards the extent of MeCP2 deficiency, which is informative for future therapeutic development.

**Electronic supplementary material:**

The online version of this article (10.1007/s13238-020-00773-z) contains supplementary material, which is available to authorized users.

## Introduction

Identifying bona fide and stable disease-relevant phenotypes in human “disease-in-dish” models is crucial for studying pathological mechanisms and developing therapeutic strategies. Rett Syndrome (RTT) is a progressive neurodevelopmental disorder, mainly caused by mutations of an X-linked gene encoding methyl-CpG-binding protein 2 (MeCP2) (Amir et al., 1999). The MeCP2 protein contains three major functional domains: a methyl CpG binding domain (MBD) that binds to 5-methylcytosine and 5-hydroxymethylcytosine to regulate chromatin structure and gene expression (Mellen et al., 2012), a transcription repression domain (TRD) proposed to recruit a co-repressor complex mSin3A and histone deacetylases (Nan et al., 1998), and a carboxyl terminal domain (CTD) that appears to interact with splicing and transcription factors, and seems to be required for MeCP2-mediated chromatin compaction (Nikitina et al., 2007). These domains are hot spots for genetic mutations associated with RTT. Eight “hotspot” mutations (R106W, R133C, T158 M, R168X, R255X, R270X, R294X and R306C) constitute more than 60% of typical cases in RTT (Neul et al., 2008). The multitude of MeCP2 mutations and random X-chromosome inactivation (XCI) often lead to rather broad variations in disease phenotypes and severities among patients (Samaco and Neul, 2011). In the past decades, genetic models for RTT in mice, particularly with MeCP2-/y male mice, have revealed some of the molecular and cellular functions of MeCP2 (Chao et al., 2007; Chahrour et al., 2008; Baudouin et al., 2012). Given that different genetic backgrounds between human and mice undoubtedly influence disease manifestations (Stettner et al., 2007), it is necessary to develop novel human cell-based RTT models, which may precisely and stably demonstrate disease-relevant phenotypes.

It has been previously reported that human embryonic stem cells (hESC) (Thomson et al., 1998) and induced pluripotent stem cells (hiPSC) (Takahashi et al., 2007), together with their relevant differentiated target cells pave the way for disease modeling, drug screening, and potential development of novel therapies (Park et al., 2008; Ebert et al., 2009; Zou et al., 2009; Marchetto et al., 2010). Though promising, there are still major confounding issues that challenge the neurological “disease-in-dish” models, e.g., how to distinguish disease relevant cellular phenotypes, and how to deal with genetic background and culture variations? Since cells in culture could be rather different from cells *in vivo*, “disease-in-dish” models are expected to capture cell intrinsic phenotypes more directly resulting from the genetic or molecular deficit, and less of those in the context of cell-cell interactions and neural circuitry functions, because it is difficult to create the cell-cell interaction context that mirrors the *in vivo* situation. To deal with the genetic background noise, one way is to derive isogenic wild type vs. diseased cells by genetic manipulations or epigenetic selections. It is also very important to establish a causal relationship between disease-related genetic mutations and tractable cellular phenotypes via gain- and loss-of-function studies with the disease genes. In addition, the genetic-phenotypic correlation needs to be validated in multiple models of the same disease, hopefully from different labs, in order to deduce consistent “disease-relevant” phenotypes so that it can be used for future therapeutic development.

Specific deletion of MeCP2 in post-mitotic neurons is sufficient to cause phenotypic impairments in mice (Luikenhuis et al., 2004). Restoration of MeCP2 expression or reactivation of the X-chromosome not only ameliorates neuronal and synaptic abnormalities, but also disease features, suggesting a tight correlation between the level of functional MeCP2 and *in vivo* or *in vitro* disease phenotypes (Przanowski et al., 2018). Both neuronal and synaptic defects have been reported in human RTT-iPSCs with various MeCP2 mutations (Marchetto et al., 2010; Li et al., 2013; Bu et al., 2017), however, the depicted disease phenotypes are variable and their correlation with MeCP2 functional states has not been fully established. In this study, by establishing isogenic wild type (WT) and mutant (MuT) RTT-iPSC lines based on X-chromosome inactivation patterns, and knockdown of MeCP2 in hiPSCs and hESCs, we revealed consistent cellular phenotypes of MeCP2-deficient neurons with different brain regional features. We also prove that the various phenotypes of MeCP2-deficient neurons including electrophysiological properties, neuronal morphology, dendritic complexity, and circuit-dependent synaptic transmissions are not only causally related to MeCP2 functional deficits but also have graded sensitivity towards the extent of MeCP2 deficiency.

## Results

### Generation of X-chromosome state-stable isogenic WT and MuT RTT hiPSCs

**Figure 1:**
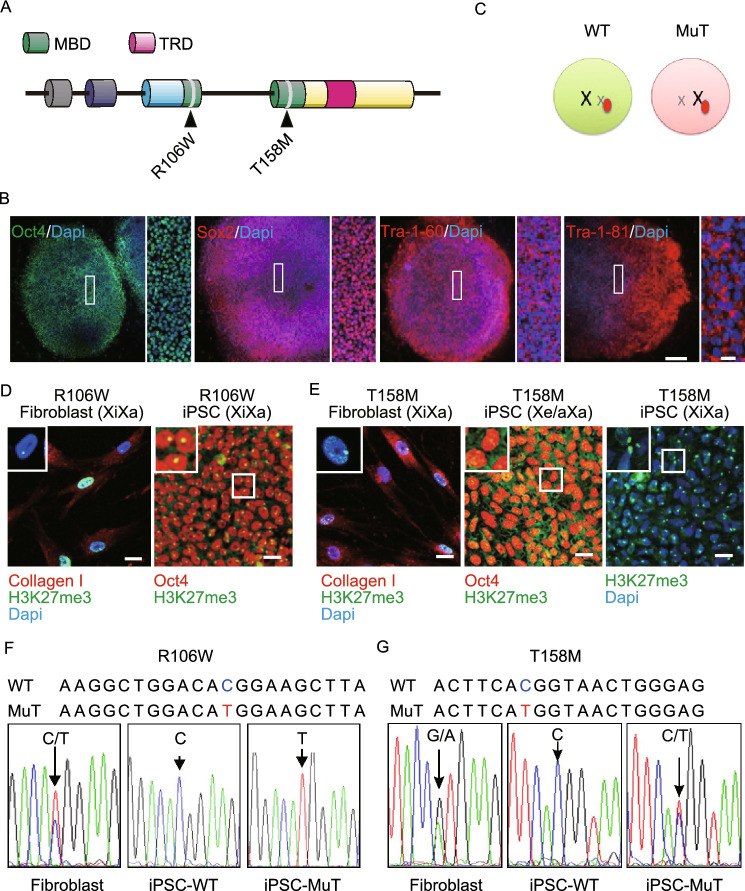
**Generation of iPSCs from RTT patient dermal fibroblasts**. (A) A schematic representing MeCP2 gene structure showing the two different point mutations analyzed in this study. MBD: methyl-CpG binding domain; TRD: transcription repression domain. (B) Representative images of RTT-iPSCs showing expression of pluripotency markers Oct4, Sox2, Tra-1-81 and Tra-1-60 as well as typical hESC colony-like morphology (Bars: 250 μm and 50 μm). (C) A schematic representing X inactivation. In the brain of RTT patients, MeCP2 mutation is chimeric due to random X inactivation. (D) Representative images of H3K27me3 immunofluorescence in RTT fibroblasts and iPSCs. All fibroblast lines exhibit predominant H3K27me3 nuclear foci (Barr body) and express fibroblast marker, collagen I. R106W iPSCs also show clear H3K27me3 foci and pluripotent marker Oct4. (E) T158M (Xa/Xa or Xa/Xe) iPSCs show diffused immune reactivity indicating the absence of Barr body (Bar: 25 μm). (F) Mono-allelic expression of MeCP2 in R106W hiPSC lines. Sequencing of MeCP2 cDNA from R106W fibroblasts showing expression of both WT and mutant MeCP2, whereas R106W iPSCs only express one of the two alleles. This is likely due to the retention of the XCI status of the individual fibroblast from which each of these hiPSC lines originates. (G) MeCP2 cDNA sequencing results from T158M fibroblasts and iPSCs showing expression of both WT and MuT MeCP2 alleles in multi clonal fibroblast cultures and in one heterogeneous (XaXe or Xa/Xa) T158M iPSC line, whereas other iPSC lines only express the WT allele

RTT patients harboring mutations in the MBD have more severe symptoms. We therefore reprogrammed 2 female RTT fibroblast lines containing R106W and T158M mutations in the MBD into iPSCs using the Yamanaka factors (Fig. 1A). RTT iPSCs expressed pluripotency markers Sox2, Oct4, Tra1-60, and Tra1-81 and maintained hESC-like morphology upon passaging for at least 42 passages with normal karyotype (46, XX), and were pluripotent as indicated by teratoma formation (Figs. 1B, S1A and S1B). Profiling of the transcriptome of our RTT-hiPSCs clustered with those of hESCs and hiPSCs acquired from public databases, but separately from those of somatic cells including neurons and fibroblasts, indicating successful reprogramming (Fig. S1C).

RTT is an X-linked disorder. Due to random XCI, all girls with RTT are almost always heterozygous for MeCP2 mutations, and patient brains contain both WT and MuT neurons. Cells with inactivation of the MeCP2 mutant allele are essentially WT, whereas cells in which the wild type allele is inactivated only express mutant MeCP2 (Fig. 1C). *In vivo*, XCI is established during early embryonic development upon differentiation of the three germ layers (Wu and Zhang, 2010), whereas in culture most human pluripotent stem cells and all somatic cells including fibroblasts have established XCI (Ananiev et al., 2011). During reprogramming, the majority of hiPSCs maintain the XCI pattern of their parental somatic cells with one transcriptionally active X (Xa) and one inactive X (Xi) (Tchieu et al., 2010; Pomp et al., 2011; Tomoda et al., 2012). Erosion of XCI (Xe) has also been observed and is a concern for X-linked disease models (Mekhoubad et al., 2012). To define the XCI status of our iPSCs, we used H3K27me3 antibody to label the Barr body, which serves as an XCI marker (Plath et al., 2003). Both R106W and T158M fibroblasts showed typical Barr body, indicating that they are properly X-inactivated (Fig. 1D and 1E). All iPSC lines from R106W fibroblasts also had Barr body (Fig. 1D), suggesting the original XCI pattern is maintained. We sequenced MeCP2 cDNAs from R106W parental fibroblasts and found double peaks, representing expression of both WT and MuT alleles in parental fibroblast cultures (Fig. 1F). Since XCI is established in these cells, based on Barr body H3K27me3 staining, we concluded that the parental fibroblasts were a mixed population of cells with either WT or MuT allele inactivated. We then sequenced MeCP2 cDNAs from R106W hiPSCs and results showed that each clone only had a single peak with either a WT or MuT pattern (Fig. 1F). Since iPSCs are most likely clonally derived, iPSC lines presented homogenous patterns of X inactivation with the expression of either WT allele or MuT allele, representing isogenic WT and MuT lines, respectively. After obtaining isogenic WT and MuT iPSC lines from R106W patient fibroblasts, we also reprogrammed T158M fibroblasts. Interestingly most T158M iPSC lines showed typical Barr Body, however, all (32/32) of the X-inactivated T158M iPSC lines that were obtained had a WT pattern of XCI, remarkably different from the parental fibroblasts, which also showed a mixed population of WT and MuT X-chromosome inactivated cells (Fig. 1E and 1G). It is therefore possible that MuT T158M cells have a reprogramming (or survival) disadvantage. Fortuitously, we also obtained one line of T158M iPSCs that showed no Barr body staining and had both MuT and WT alleles expressed, indicative of Xe or Xa (Fig. 1E and 1G). We therefore focused our study mainly on R106W RTT iPSCs, and used T158M MeCP2 expression vectors to study the function of T158M MeCP2. Our results thus demonstrated that we have generated X-chromosome inactivation state-stable isogenic RTT iPSC lines that express either WT or R106W MuT MeCP2.

### Both dorsal and ventral RTT neurons show consistent and long-lasting deficiencies in electrophysiological maturation

**Figure 2:**
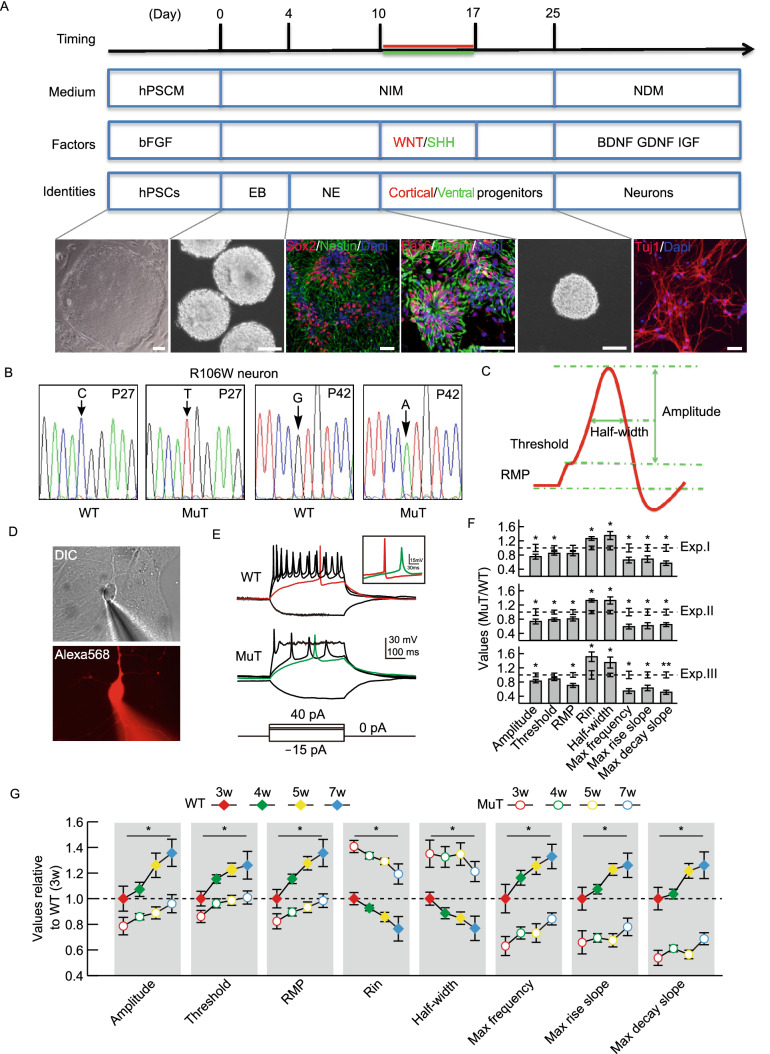
**Consistent intrinsic electrophysiological phenotypes of RTT neurons**. (A) Timeline of the neuronal differentiation protocol. Representative images show morphological changes during neural differentiation of isogenic hiPSCs. Neuro-progenitor cell (NPC) colonies, showing rosette structures, are positive for neural precursor markers Sox2, Pax6 and Nestin, whereas neurons are positive for Tuj1 (Bar: 50 μm). hPSCM, human pluripotent stem cell culture medium; NIM, neural induction medium; NDM, neural differentiation medium; hPSC, human pluripotent stem cell; EB, embryoid body; NE, neuroectoderm. (B) MeCP2 cDNA sequencing results from WT and R106W MuT neurons derived from passage 27 (P27) and passage 42 (P42) hiPSCs indicating absence of XCI erosion and X-chromosome reactivation even after 42 passages. (C) A schematic representing major parameters of active and passive membrane properties. (D) Representative DIC and fluorescence images of a hiPSC derived neuron during whole-cell recording. The lower panel shows the labeling of the neuron by infusion of Alexa 568 through the glass pipette. (E) Representative traces recorded from WT (upper panel) and MuT neurons (lower panel) in response to steps of current injection. (Bars: 30 mV and 100 ms). Insert shows the comparison of representative action potential traces from WT and MuT neurons (Bars: 15 mV and 30 ms). (F) Bar graphs showing active and passive membrane properties of MuT neurons normalized to WT neurons from three biologically independent experiments. *n* (WT1) = 43 neurons; *n* (WT2) = 47 neurons; *n* (MuT1) = 48 neurons; *n* (MuT2) = 43 neurons. Error bars stand for standard error. *P < 0.05, **P < 0.01. (G) Normalized quantification comparing active and passive membrane properties between WT and MuT neurons at four recording time points (3, 4, 5 and 7 weeks after plating on mouse astrocytes). Error bars stand for standard error. *P < 0.05

R106W WT and MuT hiPSCs were differentiated into functional neurons following a modified version of the published step-wise differentiation protocol (Fig. 2A) (Chen et al., 2016). Without extra patterning morphogens, almost all neural progenitors (NPCs) differentiated from hiPSCs expressed Pax6, Sox2 and Nestin, consistent with their forebrain dorsal identity (Fig. 2A). We did not observe any differences in neural specification efficiencies between WT and MuT hiPSCs. The efficiencies of NPC differentiation into Tuj1-positive neurons were also similar amongst all lines and were consistently close to 90% from culture to culture (Chen et al., 2016). Moreover, the XCI pattern was maintained across passages as well as after terminal neuronal differentiation (Fig. 2B).

We employed an unbiased electrophysiological approach to investigate passive and active properties of R106W WT and MuT neurons derived from hiPSCs 3–7 weeks after co-culture with astrocytes (Fig. 2C–F). We consistently observed that the intrinsic excitability was impaired in R106W MuT neurons (Fig. 2E and 2F). Mutant neurons exhibited alterations in action potential (AP) characteristics, as illustrated by smaller AP amplitude, reduced maximal firing frequency, elevated depolarization threshold, and slower action potential kinetics as indicated by prolonged half-width, decreased maximal rise and decay slopes (Fig. 2F). Passive membrane properties were also impaired in mutant neurons, as shown by more depolarized resting membrane potentials (RMP) and increased input resistance (Rin) (Fig. 2F). The observed phenotypic deficiencies in R106W MuT neurons were robust and consistent, since they were stable in various MuT iPSC lines and from batch to batch of differentiation replicates (Fig. 2F).

Given that RTT is a progressive neurodevelopmental disorder, we decided to further investigate whether these neuron intrinsic electrophysiological abnormalities exacerbate or attenuate over time. We performed whole-cell patch clamp recording on R106W WT and MuT neurons at different time points, i.e., 3, 4, 5 and 7 weeks after being plated with astrocytes. WT neurons presented a significant improvement in maturity over time, as shown by increased AP amplitude, more hyperpolarized RMP, and decreased Rin (Fig. 2G). MuT neurons, on the other hand, developed significantly slower. As a result, the differences between WT and MuT neurons in either active or passive electrophysiological properties widened over time (Fig. 2G).

**Figure 3:**
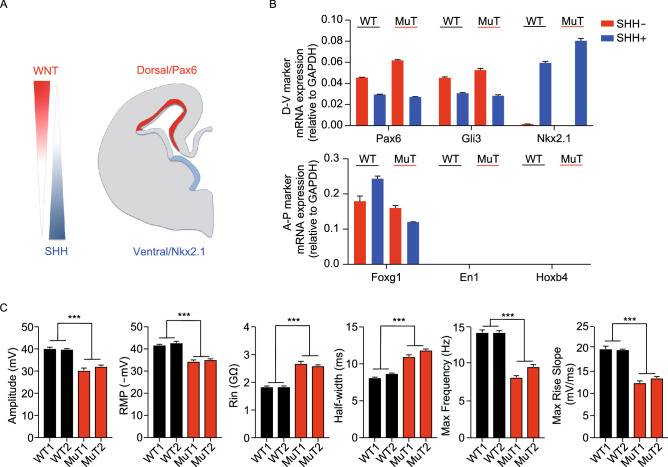
Consistent intrinsic electrophysiological phenotypes of RTT neurons after ventralization. (A) Schematic representation of dorsal/ventral and anterior/posterior identity in response to patterning morphogens of WNT and SHH. (B) Changes in the expression of dorsoventral (D-V) and but not anteroposterior (A-P) markers assessed by qPCR after ventralization of hiPSC-derived NPCs by SHH treatment. (C) Quantification comparing active and passive membrane properties between WT and MuT neurons with SHH treatment. *n* (WT1) = 24 neurons; *n* (WT2) = 22 neurons; *n* (MuT1) = 19 neurons; *n* (MuT2) = 29 neurons. Error bars stand for standard error. ***P < 0.001

To explore whether the electrophysiological deficits in RTT applies to neurons with different regional identities, we patterned WT and R106W MuT iPSCs to forebrain ventral NPCs through the usage of sonic hedgehog (SHH) (Fig. 3A) (Li et al., 2009; Liu et al., 2013; Tao and Zhang, 2016; Chen et al., 2018). In the presence of 500 ng/mL SHH from day 10–17 (Fig. 2A), the dorsal genes Pax6 and Gli3 were downregulated, and at the same time the ventral hallmark gene Nkx2.1 was robustly induced with retained forebrain gene FoxG1 expression, but not midbrain (En1) or hindbrain (Hoxb4) gene expression, suggesting efficient specification of forebrain ventral NPCs (Fig. 3B). Immunostaining experiments also showed that ventral neurons lacked Pax6 or Tbr1 expression, and mostly generated GABAergic interneurons (Fig. S2A). All these supported a complete ventralization and distinct neuronal subtypes generated in the ventral culture. Of note, we observed significant impairments in both active and passive electrophysiological properties in these ventral neurons (Fig. 3C), highlighting these intrinsic deficits are common RTT disease features, which apply for various neuronal subtypes.

### Impairments in electrophysiological properties in RTT are directly related to the dosage of functional MeCP2

**Figure 4:**
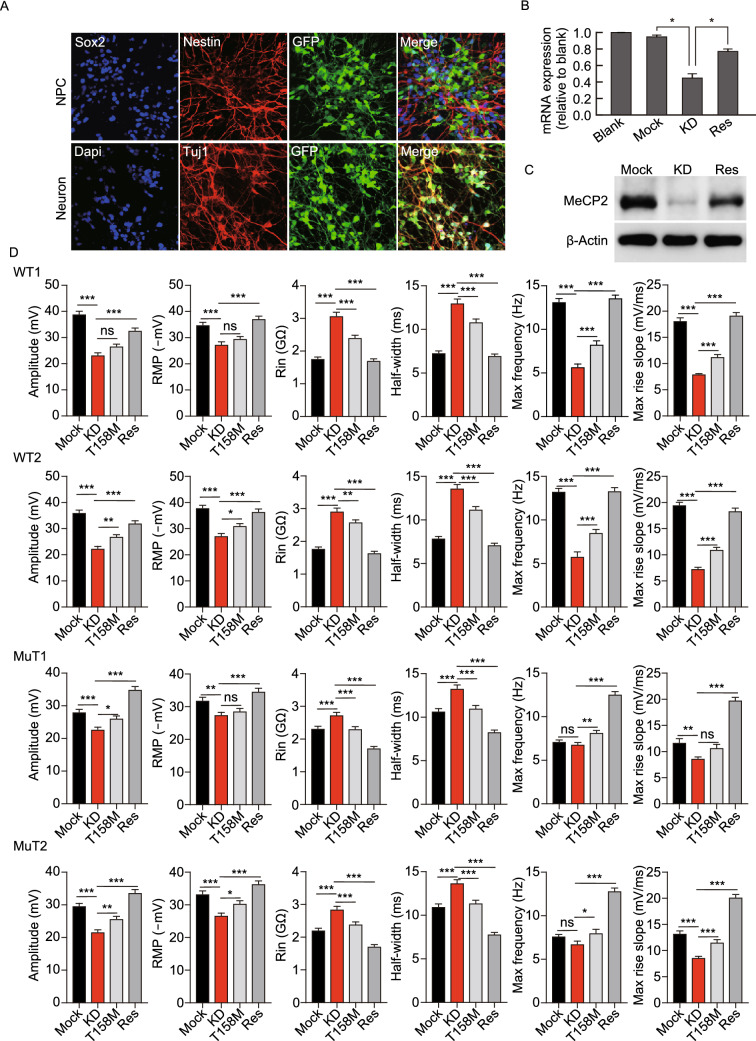
Causal relationship between MeCP2-deficiency and AP phenotypes: KD and rescue experiments. (A) Representative images of lentivirus infected hiPSC-derived NPCs, expressing Sox2 and Nestin, and neurons, expressing Tuj1, indicating continued expression of lentivirus-driven GFP during neuronal differentiation. Three types of lentivirus were used: Mock, empty lentivirus; KD, shRNA driven KD of endogenous MeCP2; Res, KD of endogenous MeCP2 and expression of exogenous shRNA resistant WT MeCP2. Every whole-cell recordings were performed on GFP labeled neurons (Bar: 25 μm). (B) MeCP2 mRNA expression assessed by qPCR in non-infected NPCs (blank) and NPCs infected with Mock, KD or rescue lentivirus. Infection with Mock lentivirus does not affect MeCP2 mRNA expression, whereas KD lentivirus reduces MeCP2 expression. Rescue lentivirus stores MeCP2 mRNA level back to normal. *n* = 3 independent experiments. *P < 0.05. (C) MeCP2 protein expression assessed by WB performed on infected NPCs showing KD of MeCP2 after infection with KD virus (KD), while rescue virus (Res) restores MeCP2 expression to a level close to WT control. (D) Electrophysiological properties of WT, R106W-MuT subjected to Mock, MeCP2-KD, MeCP2-T158M-Rescue and MeCP2-WT-Rescue. *n* (WT1-Mock) = 14 neurons; *n* (WT2-Mock) = 15 neurons; *n* (MuT1-Mock) = 13 neurons; *n* (MuT2-Mock) = 14 neurons; *n* (WT1-KD) = 8 neurons; *n* (WT2-KD) = 10 neurons; *n* (MuT1-KD) = 12 neurons; *n* (MuT2-KD) = 13 neurons; *n* (WT1-T158M) = 14 neurons; *n* (WT2-T158M) = 15 neurons; *n* (MuT1- T158M) = 14 neurons; *n* (MuT2-T158M) = 13 neurons; *n* (WT1-Res) = 15 neurons; *n* (WT2-Res)=14 neurons; *n* (MuT1-Res) = 15 neurons; *n* (MuT2-Res) = 16 neurons. Error bars stand for standard error. *P < 0.05, **P < 0.01, ***P < 0.001

To determine whether it is MeCP2-deficiency, *per se*, that caused the aforementioned neuronal electrophysiological phenotypes, we performed knock-down (KD) and rescue experiments. Three types of lentiviral vectors were used for gain- and loss-of-function studies: GFP labeled mock control; MeCP2 shRNA for KD; and shRNA KD endogenous MeCP2 with re-expression of exogenous WT MeCP2 for rescue. Under the neural differentiation conditions, we infected NPCs with these three types of viruses (Fig. 4A), respectively, and monitored MeCP2 expression levels by qPCR (Fig. 4B) and Western blot (Fig. 4C). Both analyses demonstrated effective KD of endogenous MeCP2 and that total MeCP2 protein levels in rescued neurons are similar to that of controls (WT).

Neurons differentiated from WT R106W NPCs were subjected to MeCP2 KD treatment. Compared with those in mock control groups, KD neurons exhibited impairment of AP properties and passive membrane properties, which were rescued by restoration of WT MeCP2 expression (Fig. 4D). We also repeated the KD and rescue experiments in well-studied H9 hESCs, and consistent impairments and restoration of AP and passive membrane properties were obtained in KD and rescue groups, respectively (Fig. S2B). Interestingly, R106W MuT neurons after KD of MeCP2 displayed more sever electrophysiological deficits, and full recovery of all tested electrophysiological properties were observed after restoring of WT MeCP2 expression (Fig. 4D). This indicated that R106W mutant MeCP2 was still partially functional. Moreover, the severity of the observed electrophysiological phenotypes in RTT and KD neurons were likely dosage of functional MeCP2-dependent and therefore a stable disease feature.

Since T158M mutation showed XaXe pattern of XCI after iPSC reprogramming, resulting in bi-allelic MeCP2 expression, which potentially generated a MeCP2 dosing issue, we decided to study the function of T158M MeCP2 in a different context. In MeCP2 WT and R106W MuT lines and also in the H9 hESC line, after KD of MeCP2, we ectopically expressed T158M mutant version of MeCP2, and compared that to WT rescue. The results demonstrated that T158M partially reversed the intrinsic neuronal electrophysiological properties of KD neurons (Fig. 4D). Taken together, these data suggest that MeCP2 RTT mutations, at least in the case of R106W and T158M are still partially functional, and support the idea that fully functional MeCP2 is indispensable in the development and maturation of neuronal intrinsic electrophysiological properties.

### Graded morphological deficits of RTT neurons

**Figure 5:**
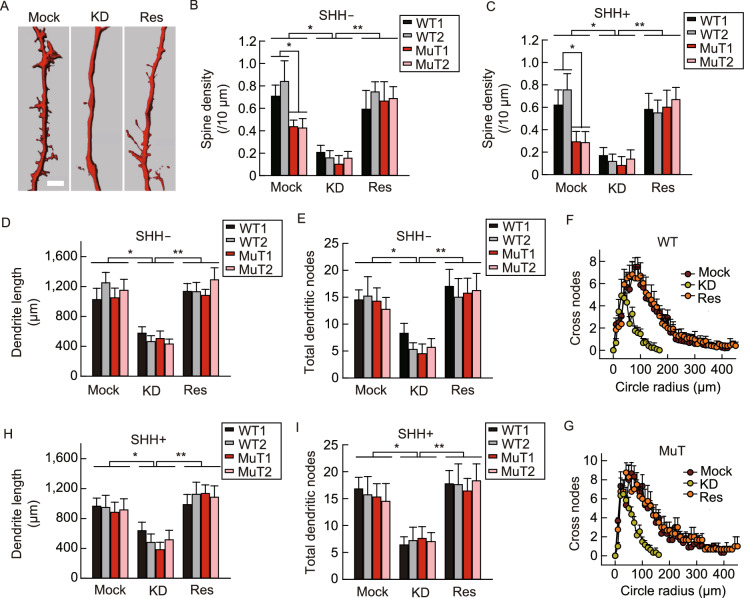
Morphological phenotype of RTT neurons. (A) Representative image showing dendritic spines from mock, KD and rescue groups (Bar: 5 μm). (B and C) Quantification of the spine density of neurons from mock, KD and rescue groups untreated (B) or treated (C) with SHH. (D) Quantification of the number of total dendritic lengths of neurons from Mock, KD and rescue groups. (E) Quantification of the number of and dendritic nodes of neurons from mock, KD and rescue groups. (F) Sholl analysis plots of WT neurons from mock, KD and rescue groups. (G) Sholl analysis plots of MuT neurons from mock, KD and rescue groups. (H) Quantification of the number total dendritic lengths of neurons from mock, KD and rescue groups treated with SHH. (I) Quantification of the number of dendritic nodes from mock, KD and rescue groups treated with SHH. For this whole figure, error bars represent standard errors. *P < 0.05, **P < 0.01, *n* = 18~30 neurons for each experiment

MeCP2 was considered to play a key role in developing and maintaining normal neuronal morphology (Calfa et al., 2011). We labeled neurons with neurobiotin included in the pipette solution during whole-cell recordings and obtained neuronal morphological features by immunostaining and subsequent 3D reconstruction of neurite and spine morphology (Fig. 5A). In R106W MuT cortical neurons, impaired spine density was clearly observed (Fig. 5A and 5B). In addition, KD of MeCP2 remarkably decreased spine density further in both WT and MuT neurons, which is reversible upon wild type MeCP2 rescue (Fig. 5B). Decreased spine density was also observed in SHH patterned ventral R106W MuT neurons, suggesting such a phenotype is pan-neuronal, but not neuronal subtype-specific (Fig. 5C).

We also investigated alterations of nuclear and soma size of MeCP2-deficient neurons. The soma size of R106W MuT neurons was significantly smaller than that of WT neurons, and both of which went through further reduction upon KD of MeCP2 (Fig. S3A–C). In great contrast, the nuclear size was comparable between WT and R106W MuT neurons (Fig. S3). However, KD of MeCP2 reduced the nuclear size in both WT and MuT groups and can both be reversed upon wild type MeCP2 expression (Fig. S3).

Similar to the results on nuclear size, the overall structures between WT and R106W MuT neurons did not show obvious difference (Fig. 5D and 5E). The dendritic length and total dendritic nodes per neuron were almost identical in WT and R106W MuT neurons (Fig. 5D and 5E). Again, KD of MeCP2 drastically decreased the overall dendritic length and the number of total dendritic nodes in both WT and R106W MuT neurons (Fig. 5D and 5E). Consistently, less complexity of dendritic morphology with KD of MeCP2 was evidently revealed by Sholl analysis (Fig. 5F and 5G). Moreover, after SHH patterning KD neurons showed similar morphological defects, but not MuT ventral neurons without KD (Fig. 5D–I). In all cases re-introducing of MeCP2 restored the overall dendritic length and the number of dendritic nodes (Fig. 5D–I).

These observations were in line with previous reports that morphological defects are typical features of RTT (Jentarra et al., 2010; Li et al., 2013). However, based on our current study, these morphological features are graded, within which deficits in soma-size and spine densities are stable and could be consistently observed in both dorsal and ventral neurons with MeCP2 mutations or with MeCP2 KD. However, deficits in dendritic complexity and nuclear size depend on a larger extension of MeCP2 loss-of-function and are only observed with MeCP2 KD but not with MeCP2 mutations.

### Synaptic transmission abnormalities occur in KD but not MuT RTT neurons

**Figure 6:**
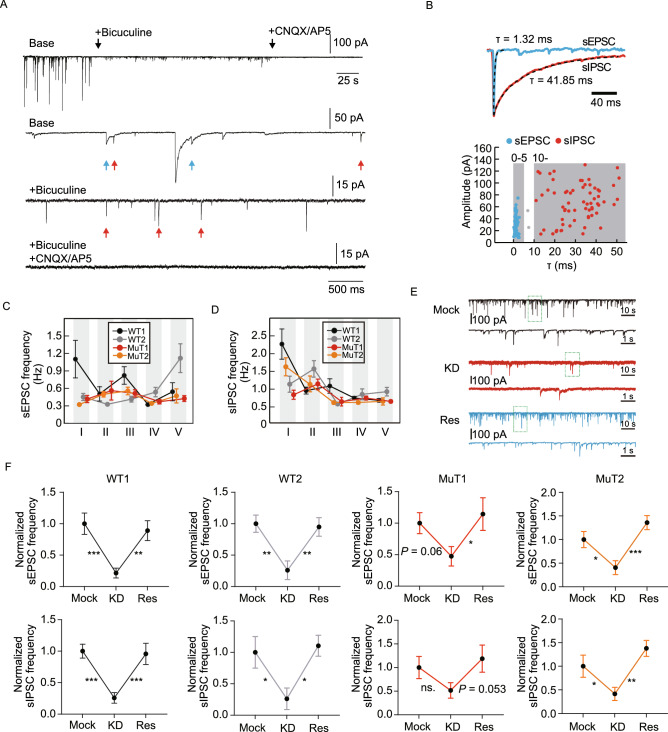
Neurotransmission phenotype of RTT neurons. (A) Representative trace showing membrane currents from a neuron subjected to application of Bicuculline (50 mmol/L), CNQX (50 mmol/L) and APV (20 mmol/L) (Bars: 100 pA and 25 s). (B) Close-up of (A) showing pharmacological isolation of sEPSCs and sIPSCs. The upper trace shows a mixture of fast-kinetics (red arrows) and slow-kinetics (blue arrows) currents. The slow-kinetics and fast-kinetics currents were sensitive to Bicuculline and CNQX/AP5 respectively, as shown in the middle and lower traces. The vertical bars represent 50, 15 and 15 pA, respectively. The horizontal bar represents 500 ms. (C and D) sEPSCs (left) and sIPSCs (right) frequencies measured in neurons from two WT (WT1/2) and R106W mutant (MuT1/2) sublines in five independent experiments. *n* (WT1) = 170 neurons; *n* (WT2) = 149 neurons; *n* (MuT1) = 144 neurons; *n* (MuT2) = 135 neurons. (E) Representative traces of sPSCs in mock (black), KD (red) and rescue (blue) groups. Lower traces are close-up of the green boxes in each upper trace. Vertical bars represent 100 pA, 10 pA and 100 pA respectively. Horizontal bars represent 10 s for upper traces and 1 s for lower traces. (F) Scatter-line plots of the frequency of sEPSC (upper panels) and sIPSC (lower panels) in KD and rescue groups normalized to corresponding Mock control groups. *n* (WT1-Mock) = 30 neurons; *n* (WT2-Mock) = 46 neurons; *n* (MuT1-Mock) = 40 neurons; *n* (MuT2-Mock) = 45 neurons; *n* (WT1-KD) = 33 neurons; *n* (WT2-KD) = 42 neurons; *n* (MuT1-KD) = 33 neurons; *n* (MuT2-KD) = 33 neurons; *n* (WT1-Res) = 38 neurons; *n* (WT2-Res) = 44 neurons; *n* (MuT1-Res) = 34 neurons; *n* (MuT2-Res) = 50 neurons. Error bars stand for standard error. *P < 0.05, **P < 0.01, ***P < 0.001

To determine whether RTT neurons with MeCP2 mutation or KD recapitulate some of the reported *in vivo* synaptic transmission defects (Asaka et al., 2006; Moretti et al., 2006; Dani and Nelson, 2009), we recorded spontaneous postsynaptic currents (sPSCs). sPSCs could be clearly classified into two groups: currents of fast kinetics (tau < 5 ms) and much slower kinetics (tau > 10 ms) (Fig. 6A and 6B). These currents with fast-kinetics were confirmed to be excitatory postsynaptic currents (sEPSCs) as they could be blocked by CNQX (50 mmol/L) or AP5 (20 mmol/L), AMPA and NMDA receptor antagonists, respectively (Fig. 6A). The slow-kinetic currents, which were sensitive to GABA_A_R antagonist Bicuculline (50 mmol/L), were proven to be inhibitory GABAergic postsynaptic currents (sIPSCs) (Fig. 6A). A few exceptions with tau between 5 to 10 ms were excluded for further analyses. Using this criterion, we performed whole-cell recording on WT and R106W MuT neurons in at least five biologically independent experiments (Fig.s 6C and 6D). Results showed that no consistent and stable differences were observed between WT and MuT groups regarding sPSCs (Fig. 6C and 6D).

On the other hand, KD of MeCP2 led to significant impairments in synaptic transmission in both WT and R106W MuT neurons after MeCP2 KD. Again, we found stable and significant deficits in sPSCs after MeCP2 KD in both WT and MuT neurons (Fig. 6E and 6F). As expected, restoring MeCP2 expression efficiently rescued both sPSCs (Fig. 6E and 6F). Thus, synaptic transmission abnormalities are high-grade RTT disease phenotypes, which could only be observed in circumstance of sever MeCP2 loss-of-function.

## Disscussion

As a neurodevelopmental disorder with autism features, RTT currently does not have any curative therapies. RTT-specific human neuronal cultures were expected to provide a powerful platform for examining the pathophysiology of RTT at molecular and cellular levels and for potential development of new drugs and therapeutic regimens (Marchetto et al., 2010; Cheung et al., 2011; Ricciardi et al., 2012). However, these “disease-in-dish” models should be carefully considered in order to fully understand the advantages and limitations of such systems so that one can use it effectively.

In this study, we established isogenic control and RTT hiPSC. Moreover, we carried out MeCP2 gain- and loss-of-function studies to determine the causality between MeCP2 deficiency and neuronal phenotypes in both RTT hiPSCs and hESCs. Using several independent models, we identified impairments in neuronal development as indicated by immature AP and altered passive membrane properties, reduced dendritic spine densities and soma sizes, a series of consistent, stable and RTT-relevant phenotypes. Moreover, these highly stable disease phenotypes are likely also pan-neuronal, because they are shared by different neuronal subtypes-derived from progenitors of either dorsal or ventral forebrain features. These results demonstrate some of the utilities of using iPSC and ESC-derived models to study complex brain disorders including RTT (Chapleau et al., 2009; Farra et al., 2012).

On the other hand, we consistently observed discrepancies between KD-based and MeCP2 MuT-based disease models when neuronal dendritic complexity, nuclear sizes, and neural network-dependent sPSCs were analyzed. KD of MeCP2 always ended up with more severe disease phenotypes as compared to MeCP2 mutations; KD of MeCP2 in R106W MuT neurons further exacerbated their electrophysiological abnormalities, suggesting that R106W version of MeCP2 is still partially functional. Similarly, knockdown endogenous and concomitantly express ectopic T158M mutant MeCP2 in WT, R106W MuT, and H9 hESCs could also partially rescue the disease phenotypes, indicative of partial function of T158M mutation. Taken together, amongst the various phenotypes observed for neurons with MeCP2-deficiency, AP, passive membrane properties, soma-sizes, and spine densities are very robust and stable, which can be consistently observed either with MeCP2 mutations or with MeCP2 KD. On the other hand, phenotypes in dendritic complexity, nuclear size, and synaptic transmission, could only be consistently observed with MeCP2 knockdown but not with MeCP2 mutations, indicative of graded sensitivities of different phenotypes towards MeCP2 deficiency.

Although mouse and monkey whole animal models have been established for RTT, human ESC and iPSC-based “disease-in-dish” models still have inimitable value. It has the advantages of harboring the most relevant genetics, easily genetically manipulatable, easily scalable, and suitable for molecular mechanistic studies. It is also easily adaptable to large-scale high through-put drug screening if appropriate targets were identified. Taken together, the seemly finicky neurological “disease-in-dish” models, if done properly, and in combination with non-human primate disease models, could make significant contributions to understanding disease pathophysiology as well as discovery of drug targets and development of therapies.

## Materials and methods

### Cell culture

RTT patient fibroblast cell lines were obtained from the Coriell Institute for Medical Research (GM11273 for R106W and GM17880 for T58M). RTT hiPSCs were generated by transduction of Oct4, Sox2, Klf4 and c-Myc through retroviral infection as described (Takahashi et al., 2007). After 3 weeks induction, hESC-like colonies were manually picked and expanded for further characterization by AP staining (data not shown). RTT hiPSCs and H9, obtained from the WiCell Institute, were cultured on irradiated mouse embryonic fibroblasts (MEFs). hiPSC/hESC cultures were regularly monitored for expression of pluripotent markers by immunofluorescence. NPCs and neurons were obtained following a step-wise differentiation protocol described previously (Zhang et al., 2001; Li et al., 2009; Liu et al., 2013; Chen et al., 2016; Tao and Zhang, 2016). The astrocytes were generated from C57BL/6. Briefly, newborn mouse cortex was dissected in chilled HBSS with careful strip of meninges. Tissue was digested in HBSS containing 0.05% trypsin and DNase and was dissociated by using a fire-polished Pasteur glass pipette and plated onto 10 cm dish. Upon reaching confluence, glial cells were trypsinized and passaged 3 times to remove neurons before the primary astrocyte co-cultures with human neurons.

### Immunostaining and Western blot (WB) analysis

Immunostaining and WB were performed as described (Chen et al., 2019). The following antibodies were used: mouse anti-Oct4 (Millipore), goat anti-Sox2 (Santa Cruz), mouse anti-Tra1-81 (Millipore), mouse anti-Tra1-60 (Millipore), mouse anti-Nestin (Chemicon), rabbit anti-Tuj1 (Covance), mouse anti-Tuj1 (Sigma), mouse anti-MAP2 (Sigma), rabbit H3K27me3 (Santa Cruz), rabbit anti-Pax6 (Covance), mouse anti-GABA (Sigma), mouse anti-Nkx2.1 (Millipore), rabbit anti-Tbr1 (Chemicon), mouse anti-vGluT1 (Millipore). MeCP2 antibody used in Western blot was a gift from Dr. Keping Hu.

### RNA extraction and quantitative real-time PCR

Total RNA was extracted from cultured cells using TRIzol (Invitrogen). After phenol/chloroform extraction and DNase (Ambion, TURBO DNA-free Kit) treatment, cDNA was generated from 1 μg of total RNA using Super-Script III First-Strand Synthesis System (Invitrogen). Quantitative real-time PCR were performed using Power SYBR Green PCR Master Mix and AB7500 system (Applied Biosystems). Pax6 forward, 5′-TCTTTGCTTGGGAAATCCG-3′; Pax6 reverse, 5′- CTGCCCGTTCAACATCCTTAG-3′; Gli3 forward, 5′- TTGCACAAAGGCCTACTCGAGACT-3′; Gli3 reverse, 5′- CTTGTTGCAACCTTCGTGCTCACA-3′; Nkx2.1 forward, 5′- AACCAAGCGCATCCAATCTCAAGG-3′; Nkx2.1 reverse, 5′- TGTGCCCAGAGTGAAGTTTGGTCT-3′; FoxG1 forward, 5′- AGAAGAACGGCAAGTACGAGA-3′; FoxG1 reverse, 5′- TGTTGAGGGACAGATTGTGGC-3′; En1 forward, 5′- GGACAATGACGTTGAAACGCAGCA-3′; En1 reverse, 5′- AAGGTCGTAAGCGGTTTGGCTAGA-3′; Hoxb4 forward, 5′- AAAGAGCCCGTCGTCTACC-3′; Hoxb4 reverse, 5′- GTGTAGGCGGTCCGAGAG-3′; Gapdh forward, 5′- GAAGGTGAAGGTCGGAGTC-3′; Gapdh reverse, 5′- GAAGATGGTGATGGGATTTC-3′.

### Teratoma formation

RTT hiPSCs were injected subcutaneously to NOD-SCID mice. Six weeks after injection, teratomas were dissected. Sections were stained with hematoxylin and eosin. All animal experiments were performed according to the protocols approved by the Institutional Animal Care and Use Committee.

### Karyotyping analysis

Standard G-banding chromosome analysis was performed by Tongji Hospital.

### Electrophysiological recording and analyses

Whole-cell patch clamp recordings were carried out on hiPSC/hESC-derived neurons co-cultured with astrocytes for 3 to 7 weeks. The neuronal density is 20,000/well (24 well plate). Recordings were made using Multiclamp 700B amplifier (Molecular Devices). The bath was constantly perfused with fresh artificial cerebrospinal fluid (ACSF) at room temperature. The ACSF contained (in mmol/L) 126 NaCl, 3 KCl, 1.25 KH_2_PO_4_, 1.3 MgSO_4_, 3.2 CaCl_2_, 26 NaHCO_3_, and 10 glucose, bubbled with 95% O_2_/5% CO_2_. Signals were sampled at 10 kHz with a 2 kHz low-pass filter. The whole-cell capacitance was fully compensated. Recordings with Ra > 50 MΩ or fluctuation >20% were excluded. Recording of intrinsic and extrinsic membrane properties were carried out at voltage-clamp mode. The intracellular solution contained followings (in mmol/L): 126 K-gluconate, 4 KCl, 10 HEPES, 4 ATP-Mg, 0.3 GTP-Na_2_, 10 creatine phosphate, 0.5% Alexa Fluor 568 hydrazide (Invitrogen) (pH 7.2, 290/300 mOsm), and 0.4% neurobiotin (Invitrogen). Membrane potentials were hold around −70 mV, and step currents with an increment of 3 pA were injected to elicit action potentials. Spontaneous postsynaptic currents were recorded from cells clamped at −70 mV with the intracellular solution containing (in mmol/L): 20 CsCl, 100 cesium methanesulfonate, 10 HEPES, 4 ATP-Mg, 0.3 GTP-Na_2_, 10 creatine phosphate, 10 QX-314, 0.5% Alexa Fluor 568 hydrazide (Invitrogen) (pH 7.2, 290/300 mOsm) and 0.4% neurobiotin (Invitrogen). Clampfit software was used to measure active and passive membrane properties. Minianalysis software was used to detect and measure excitatory and inhibitory spontaneous postsynaptic currents.

### Morphological analyses

Recorded cells were filled with neurobiotin present in the intracellular solution via positive current injection (400 pA for 250 ms, 1.25 Hz) for at least 6 min. After recording, cover slips were fixed in 4% PFA in 0.01 mol/L PBS for 10 min then rinsed with 0.01 mol/L PBS. Neurobiotin labeled cells were visualized via overnight incubation with 1:500 Cy3-conjugated streptavidin (Jackson Immuno Research Laboratories). Serial Z-stack images were taken on an Olympus FV1000 confocal system. Whole cell morphological reconstruction and analyses were carried out with Neurolucida software.

### Statistical analysis

Unless explicitly stated, all data are presented as mean ± SEM. For experiments in which only two groups were analyzed, the *t*-test was used. Differences between groups were evaluated by one-way ANOVA test with *post hoc* Tukey’s multiple comparisons tests. *P*-value of <0.05 was considered statistically significant.

## Electronic supplementary material

Below is the link to the electronic supplementary material.

Electronic supplementary material 1 (DOCX 8096 kb)
