# Supplementary material for: Graded and pan-neural disease phenotypes of Rett Syndrome linked with dosage of functional MeCP2
Source: Protein Cell. 2020 Aug 27;12(8):639–52. doi: 10.1007/s13238-020-00773-z (PMC8310543; doi:10.1007/s13238-020-00773-z)

**Supplementary Figure 1. Generation and characterization of iPSCs from RTT patients**

(A) Representative images of teratomas formed from isogenic WT and R106W MuT hiPSCs in SCID mice showing formation of tissues related to the three germ layers, ectoderm (neuronal rosettes), mesoderm (muscle and bone) and endoderm (epithelium).

(B) Representative images of normal karyotypes obtained from WT and R106W MuT iPSCs and H9 hESCs at passage 27.

(C) Analysis of the global molecular signature of RTT-iPSCs indicating successful reprogramming. Heatmap and dendrogram of hierarchical clustering (Pearson correlation) over whole genome gene expression between R106W hiPSCs, H9 hESCs, human dermal fibroblasts and hiPSC/hESC-derived neurons. hiPSC and hESC samples cluster together and separately from neurons and fibroblasts demonstrating successful reprogramming of RTT hiPSCs.

**Supplementary Figure 2. Knockdown of MeCP2 causes electrophysiological impairments in neurons derived from H9 hESCs**

(A) hESCs were differentiated toward dorsal or ventral neural progenitors by patterning in the absence or presence of SHH, respectively. Neural progenitors were then plated down on culture surfaces for neurogenesis for 1 day (day 25 post differentiation), 6 days (day 30 post differentiation), and two weeks (day 38 post differentiation). Neuronal identities were characterized by immunostaining for Pax6, Nkx2.1, Tbr1, Tuj1, vGluT1 and GABA. Scale bar, 50 μm.

(B) Action potential properties and passive membrane properties of H9 neurons subjected to Mock, MeCP2-KD and Rescue treatments. ** p<0.01, ***p<0.001, n.s.: no significance.

**Supplementary Figure 3. Changes in soma and nuclear sizes in MeCP2-deficient neurons**

(A) Representative images showing nucleus (Hoechst) and soma (MAP2) of R106W-WT and -MuT derived neurons subjected to Mock, MeCP2-KD or Rescue treatment. Scale bar, 10 μm.

(B) Distributions of soma size (left panel) and nuclear size (right panel) of R106W-WT, and -MuT neurons subjected to Mock, MeCP2-KD or rescue treatment.

(C) Box plots showing soma size (left panel) and nuclear size (right panel) of R106W-WT, and -MuT neurons subjected to Mock, MeCP2-KD or rescue treatment. KD of MeCP2 severely reduced the soma sizes. Restoration of wild type MeCP2 rescued the phenotype. a.u., arbitrary unit. Error bars stand for standard error. * p<0.05.


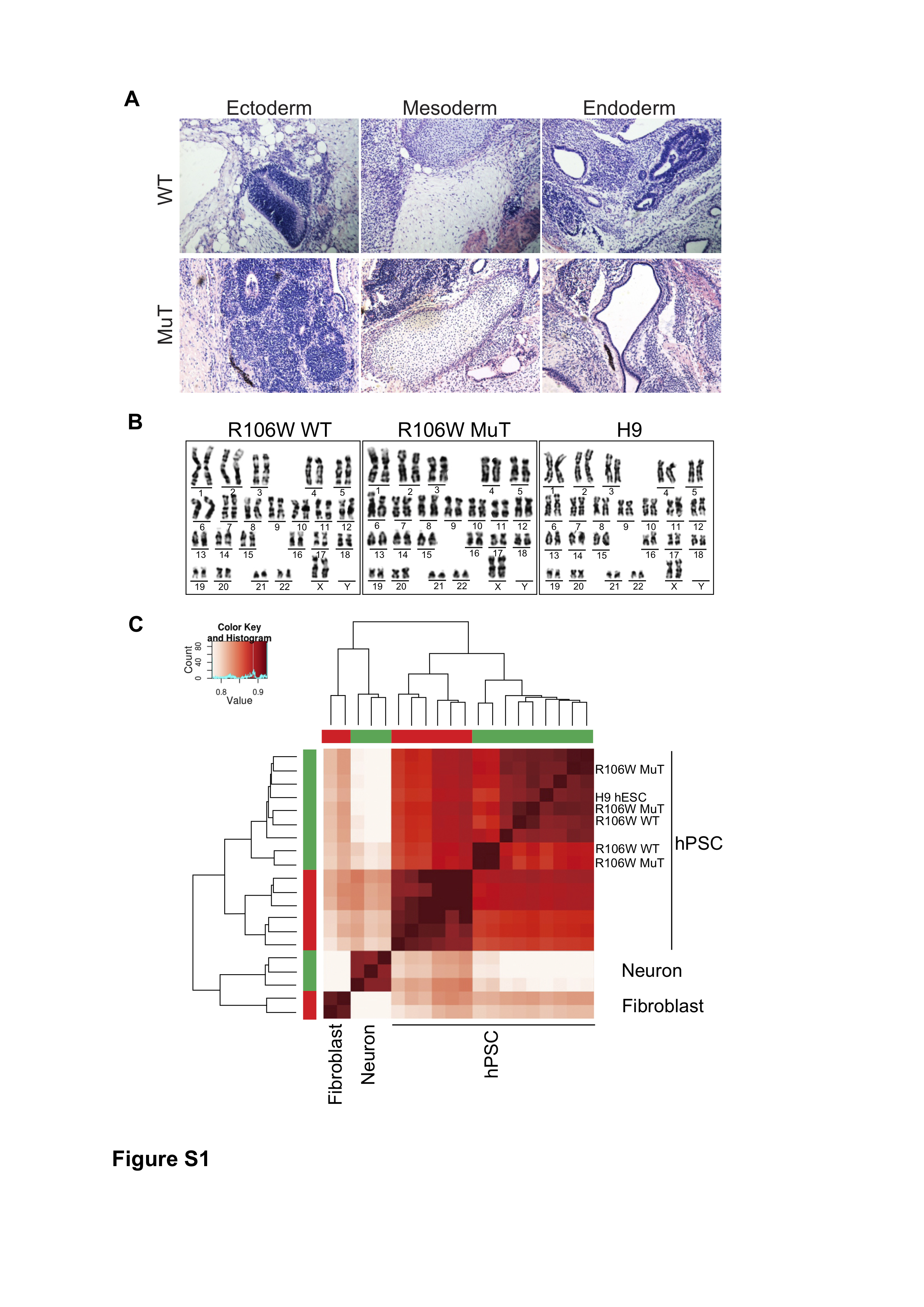


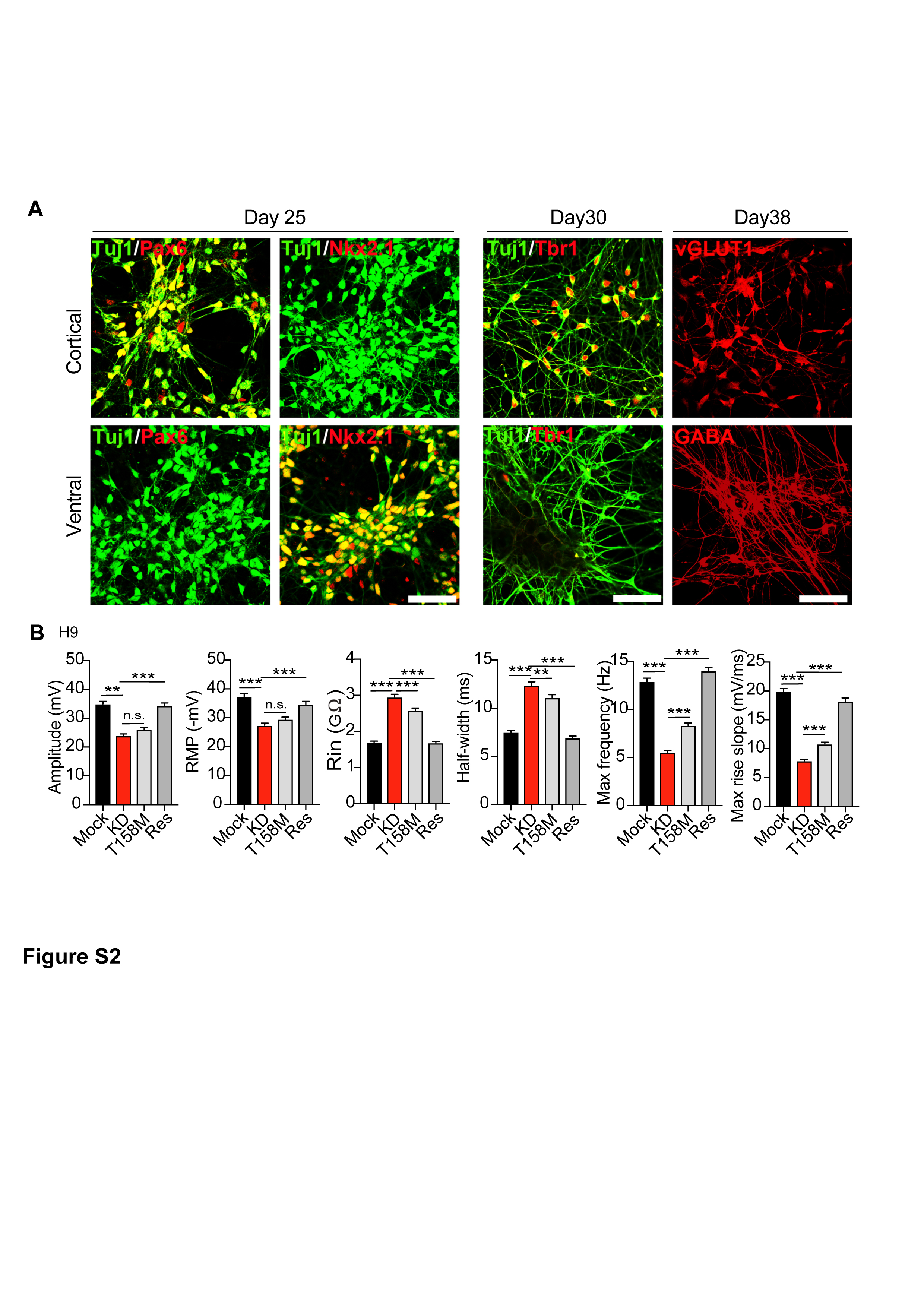


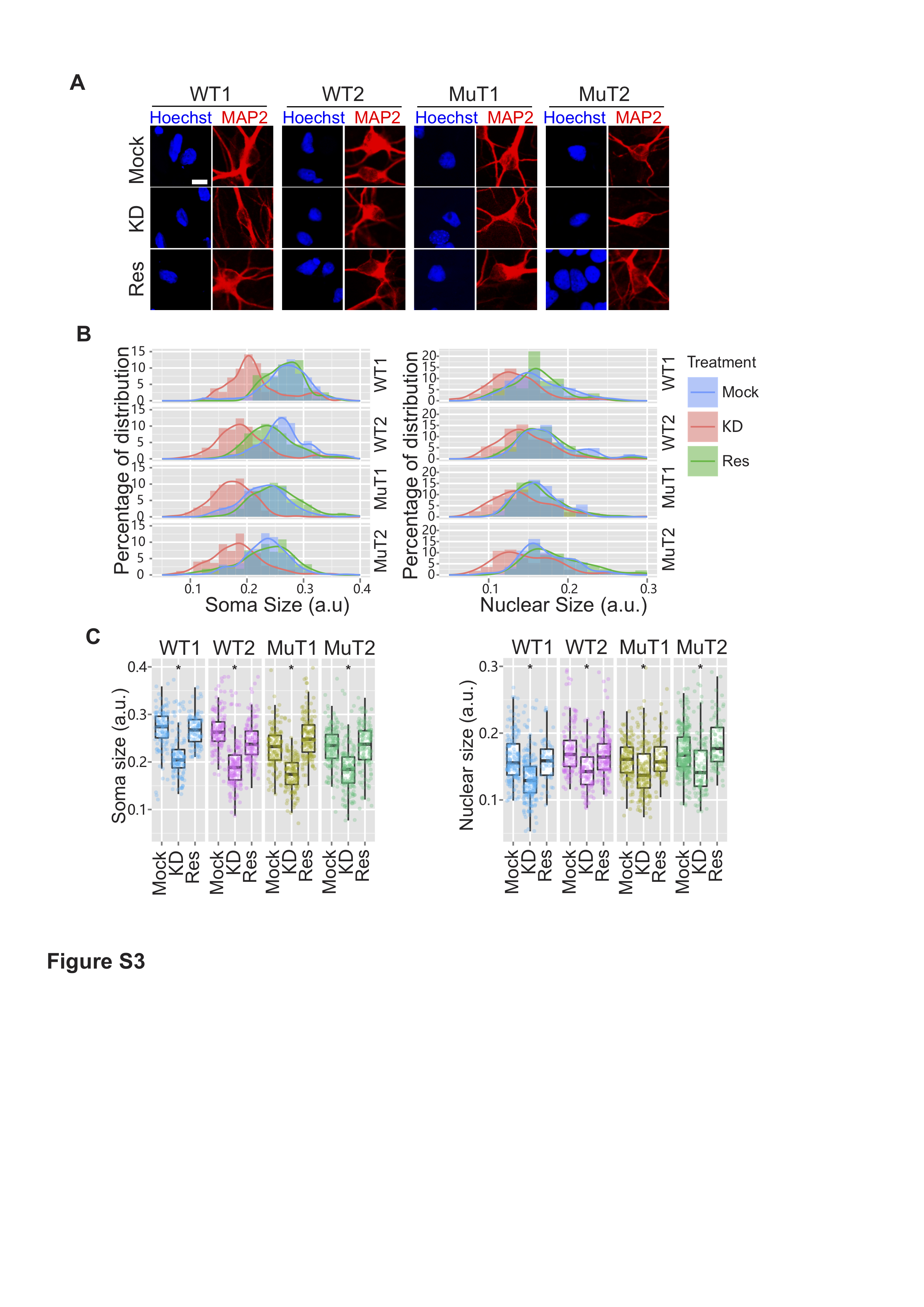

Supplement: Supplementary file 1 — Electronic supplementary material 1 (DOCX 8096 kb) [file 13238_2020_773_MOESM1_ESM.docx]
